# *In Vivo* Laser Scanning Confocal Microscopy of Human Meibomian Glands in Aging and Ocular Surface Diseases

**DOI:** 10.1155/2016/7432131

**Published:** 2016-03-07

**Authors:** Vincenzo Fasanella, Luca Agnifili, Rodolfo Mastropasqua, Lorenza Brescia, Federico Di Staso, Marco Ciancaglini, Leonardo Mastropasqua

**Affiliations:** ^1^Ophthalmology Clinic, Department of Medicine and Aging Science, “G. d'Annunzio” University of Chieti-Pescara, 66100 Chieti, Italy; ^2^Ophthalmology Unit, Department of Neurological, Neuropsychological, Morphological and Movement Sciences, University of Verona, 37126 Verona, Italy; ^3^Ophthalmic Clinic, Department of Life, Health and Environmental Sciences, University of L'Aquila, 67100 L'Aquila, Italy

## Abstract

Meibomian glands (MGs) play a crucial role in the ocular surface homeostasis by providing lipids to the superficial tear film. Their dysfunction destabilizes the tear film leading to a progressive loss of the ocular surface equilibrium and increasing the risk for dry eye. In fact, nowadays, the meibomian gland dysfunction is one of the leading causes of dry eye. Over the past decades, MGs have been mainly studied by using meibography, which, however, cannot image the glandular structure at a cellular level. The diffusion of the* in vivo* laser scanning confocal microscopy (LSCM) provided a new approach for the structural assessment of MGs permitting a major step in the noninvasive evaluation of these structures. LSCM is capable of showing MGs modifications during aging and in the most diffuse ocular surface diseases such as dry eye, allergy, and autoimmune conditions and in the drug-induced ocular surface disease. On the other hand, LSCM may help clinicians in monitoring the tissue response to therapy. In this review, we summarized the current knowledge about the role of* in vivo* LSCM in the assessment of MGs during aging and in the most diffuse ocular surface diseases.

## 1. Introduction

Meibomian glands (MGs) are holocrine glands embedded in the tarsal plate of the eyelids. Each gland comprises multiple acini connected by a long common central duct running throughout the entire length of the gland [1]. The functional unit of a meibomian gland is the meibocyte, which synthesizes the meibum, a lipoid complex forming the superficial layer of the tear film. Meibum permeates the tear surface where it serves several important functions: it prevents tear evaporation and desiccation of the ocular surface, acts as a physical and hydrophobic barrier to the inward movement of environmental and organic agents, lubricates the ocular surface preventing irritation, and promotes clear ocular vision due to its optic properties. Thus, tear physiology is dependent upon the proper functioning of the MGs [2, 3].

Meibography represented for many years the only diagnostic approach to observe the MGs morphology* in vivo* [4].

With the recent diffusion of the laser scanning confocal microscopy (LSCM) (HRT I–III Rostok Cornea Module (RCM), Heidelberg Engineering, Heidelberg, Germany), it was possible to study the microscopic anatomy of several adnexal and ocular surface structures such as eyelid, conjunctiva, cornea, limbus, tarsal plate, and MGs [5–12]. LSCM allows integrated high-resolution evaluation of the morphofunctional ocular surface unit in normal and pathological conditions [13]. Several conditions may affect the anatomy and function of MGs, such as the primary meibomian gland dysfunction, dry eye, ocular allergy, the use of contact lenses, and the long-term application of topical medications in chronic ocular surface diseases and in glaucoma. In this review, we discuss the main MGs modifications during aging and during the course of the most diffuse ocular surface diseases.

## 2. Methodology and Results

PubMed searches were performed on September 20, 2015, using the following phrases: “*in vivo* confocal microscopy” and “meibomian glands”, which identified thirty-four unique publications. Six were excluded, since they did not concern the use of IVCM to study MGs.

Three of the considered publications were not published in English; however, they provided enough information in the English abstract to warrant inclusion.

### 2.1. Meibomian Glands in the Healthy and Aging Human Eye

The confocal images are captured from a coronal scan through a mass of acini posterior to a central, vertically oriented MG duct [14]. LSCM provides structural and functional information of the glandular status: MGs acini appear to be constituted by convoluted borders with large cells lining the acini and fine cellular material within the lumen [14].

The main structural modifications induced by aging were evidenced for the first time by Wei et al. [15] and afterwards confirmed by Villani et al. [16] in a heterogeneous population study, which revealed significant negative correlations between age and meibomian glandular acinar unit density [1]. LSCM permits indirect functional evaluation of MGs, by assessing the secretion reflectivity [4].

**Figure 1:**
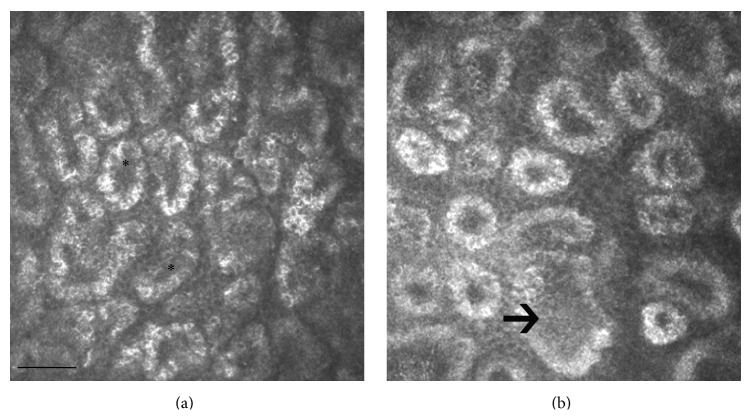
LSCM of MGs in two healthy Caucasian subjects. (a) A 26-year-old male with a normal feature and density of the acini (asterisks); (b) a 73-year-old male with decreased MG acinar density and increased acinar size (arrow), indicative of atrophic age-related changes. Bar represents 50 *µ*m.

Besides the acinar density reduction, the normal aging induces also an evident decrease of the acinar diameter (Figure 1) and an increase of the acinar wall inhomogeneity, without significant modifications of the glandular orifice diameter. The overall interpretation of the structural modification suggests glandular dropout with qualitative changes of the meibum. Therefore, the authors speculated that acinar atrophy leads to a decrease in the MGs secretion with aging. These results are in accordance with the main clinical changes observed during aging, represented by tear film break-up time (BUT) and Schirmer test scores reduction.

Even though several studies reported likely hormone-dependent changes of MGs structure [17–19], LSCM did not document significant structural and functional differences between genders during aging [19, 20]. Further studies are warranted to elucidate the clinical implications of the hormonal action on MGs age-related changes.

In summary, LSCM in healthy subjects provides information of the normal modifications of glands with aging. Normal aging induces atrophic involution of the glandular unit along with progressive dysfunction of the secretive activity (Table 1). This appears to be in line with the involution of most parts of secretive structures, such as exocrine glands and lymphatics, observed in several tissues of the human body during aging [21–23]. In the eye, the glandular unit involution may take part in the increased risk of dry eye related conditions observed in the elderly.

### 2.2. Primary and Secondary Meibomian Glands Dysfunction

The primary meibomian gland dysfunction (MGD), characterized by inflammatory changes of the lid margin structures and in the anatomy of the MG orifices, is one of the most common ocular surface disorders: Hom et al. [20] reported prevalence of 38.9%, whereas Stanek [41] reported prevalence of 71.7% in individuals above 60 years old. MGD is a major cause of dry eye and results in a qualitative alteration and/or a quantitative reduction of the lipid secretion, which leads to decreased tear stability, increased tear evaporation, loss of lubrication, and damage to the ocular surface epithelia. LSCM, which currently has a primary role in the ocular surface analysis [26, 27], allows studying MG at cellular level. In MGD, LSCM permits the analysis of the cellular density of the superficial and basal epithelium of the eyelid and the assessment of the mean acinar area and density of MGs, the glandular orifice area, the meibum secretion reflectivity, and the inflammatory features of periglandular interstices and acinar unit wall (patterns of inhomogeneity) [40]. In the first confocal study on MG diseases, Messmer et al. [28] showed dilatation and obstruction of the meibomian gland ducts in twelve patients with blepharitis/meibomitis or MGD. On the other hand, in fifteen out of 19 patients with blepharitis/meibomitis, but not in MGD, intense inflammation was observed in the tarsal conjunctival epithelium and in the stroma. The inflammatory reaction was defined by an increase of hyperreflective roundish elements corresponding to immune cells. Matsumoto et al. [29] reported that the mean acinar unit density was significantly lower in MGD patients than in control subjects, whereas the mean acinar unit diameter was significantly larger in MGD patients than in controls. Both the density and diameter of MGs acinar units significantly correlated with the severity of MGs dropout and expression grades (Figure 3).

Other confocal parameters that presented acceptable sensitivity and specificity for the diagnosis of MGD were the longest and shortest MG diameters: in the study of Ibrahim et al., these parameters resulted to be significantly worse than those observed in the controls [30]. As stated above, LSCM can also provide information about the inflammatory status of MG during MGD. Matsumoto et al. [42] measured the mean inflammatory cell density (cells/mm^2^) from the periglandular site of the entire lower and upper eyelid, reporting inflammatory cell numbers approximately ten to thirty times higher than those found in healthy controls. Interestingly, these cells markedly reduced after medical therapy: the combination of lid hygiene, topical nonpreserved artificial tears with 0.1% sodium hyaluronate, topical 0.5% levofloxacin and 0.1% fluorometholone, and oral minocycline (100 mg twice a day for 12 weeks) significantly improved the tear stability, the fluorescein staining scores, and the lid injection and cleared periglandular inflammatory infiltrates.

In a very recent study, LSCM revealed an increased immune cell number in the palpebral conjunctiva of patients with refractory MGD, compared to patients with therapy-responding MGD [43]. In the therapy-responding group, the mean inflammatory cell density in the periglandular area reduced from baseline values of 1216 ± 328 to 700 ± 436 cells/mm^2^ at the last follow-up. No statistically significantly difference was found in the group that did not receive any therapy, 882 ± 301 cells/mm^2^ before the initiation of trial and 843 ± 321 cells/mm^2^ at the final follow-up.

Interestingly, OSDI scores correlated with epithelial immune cells infiltrating the palpebral conjunctiva, suggesting that the inflammation of the palpebral conjunctiva may contribute to explaining the MGD-associated refractory symptoms.

**Figure 2:**
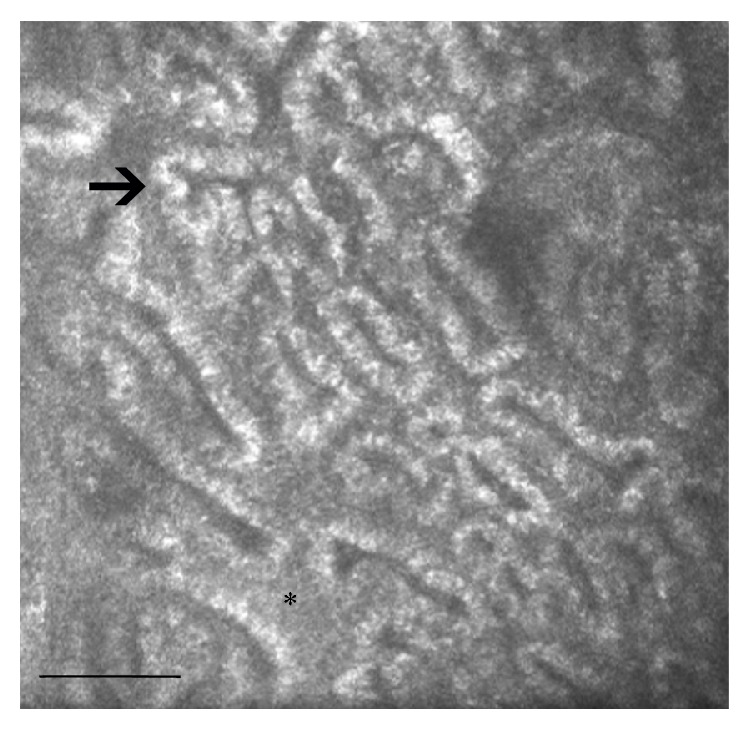
LSCM of MGs in a contact lens wearer. The most evident modifications are represented by the inhomogeneity of periglandular interstices (asterisk) and MG wall (arrow), periglandular inflammation, and the reduction of MG duct diameters. Bar represents 50 *µ*m.

**Figure 3:**
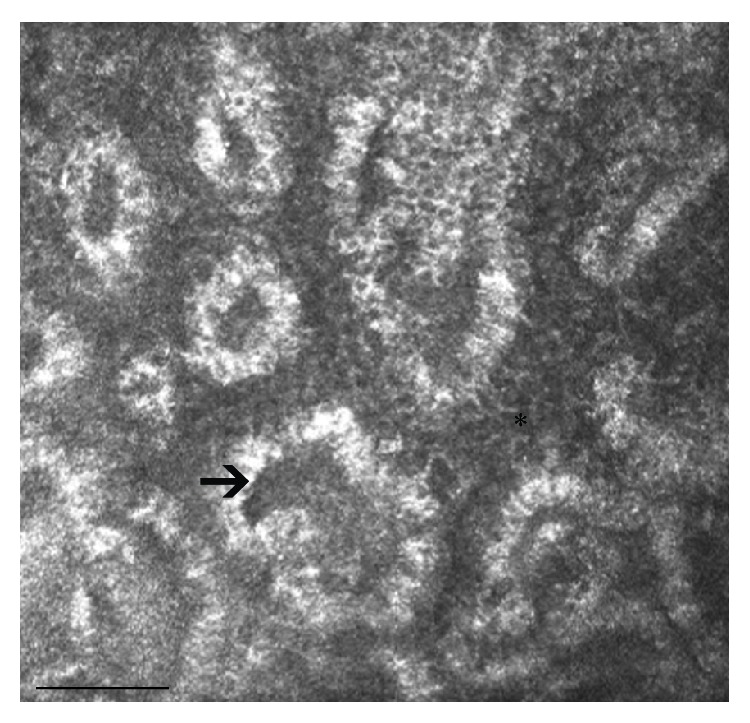
Confocal microscopy features in a 47-year-old male patient with MGD: inhomogeneity of periglandular interstices (asterisk) and MG wall (arrow), dilation and obstruction of the MG ducts, and reduction of the density of MG acinar units are the most significant alterations. Bar represents 50 *µ*m.

Secondary MGD can develop as a complication of the use of topical medications in patients with glaucoma [44–46] and in contact lens wearers (CLWs) (Figure 2) [32].

In CLWs, a decreased basal epithelium cell density, reduced acinar unit diameters, higher glandular orifice diameters, greater secretion reflectivity, and greater inhomogeneity of the periglandular interstices were the main observed findings. Morphologic changes in the MGs shown by LSCM were interpreted by the authors as signs of MGs dropout, duct obstruction, and glandular inflammation caused by chronic mechanical contact lens irritation.

In summary, MGD has a huge impact on the daily clinical practice since it plays a major role in the development of dry eye related conditions. Confocal microscopy precisely depicts, at a cellular level, the main macroscopic and microscopic MGs changes in patients affected with both primary and secondary MGD. The main features are represented by the MGs dropout, which is the glandular unit loss, the increase of the acinar surface (except for glaucoma), which, along with the increased meibum viscosity, is the hallmark of the glandular malfunction, and the inflammation of the periglandular interstice and acinar wall. The dilation of the duct orifice and the increased acinar diameter are the compensatory mechanism adopted by the glands to overcome the meibum stagnation (Table 1). All these changes lead to a decrease of the meibum production, which negatively affect the tear film stability and induce dry eye.

Therefore, MGD could be in part intended as accelerated aging of the glands, in which there is a marked inflammatory and immune component. Also, these aspects are shared by other exocrine glands' dysfunction in the human body, such as salivary glands [21–23].

In this field, confocal microscopy may provide a significant advancement in the daily clinical practice since it helps clinicians in the early diagnosis of primary or secondary MGD and in monitoring the response to therapy and the side effects of drugs.

In fact, as recently reported in a study that evaluated the efficacy of wet chamber warming goggles (Blephasteam®) in MGD patients unresponsive to warm compress treatment, LSCM documented a decreased acinar diameter and area, which was associated with an increased OSDI score [38].

### 2.3. Meibomian Glands in Dry Eye

**Figure 4:**
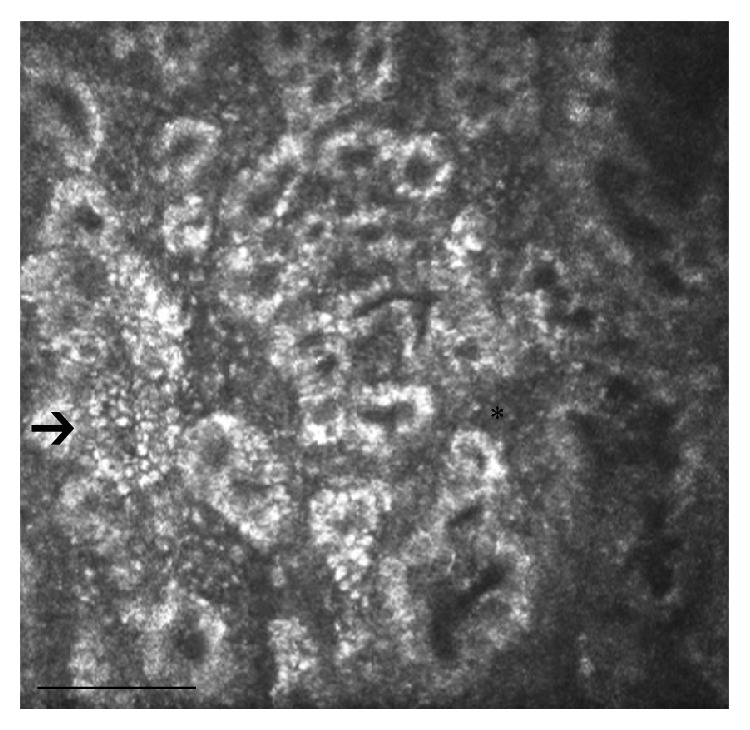
LSCM of MGs in a patient with dry eye: the image shows a decreased diameter of meibomian gland ducts, along with marked inhomogeneous appearance of the MG walls and interstices. Several punctate hyperreflective elements, which reflect a high degree of local inflammation, are recognizable in the interstice and within the MG wall (arrow and asterisk). Bar represents 50 *µ*m.

Dry eye syndrome (DES) is one of the most common disorders of the eye, with prevalence of 10% to 20% in the adult population. The International Dry Eye Workshop (2007) [47] defined DES as a “multifactorial disease of the tears and ocular surface that results in symptoms of discomfort, visual disturbance, and tear film instability with potential damage to the ocular surface. It is accompanied by increased osmolarity of the tear film and inflammation of the ocular surface.” In this field, LSCM documented the fine and peculiar microscopic findings of the target tissues including the cornea, conjunctiva, and meibomian glands. While several reports studied the confocal corneal and conjunctival changes in DES, MGs were only partially studied. In patients with primary DES and MGD, Villani et al. [33] reported increased acinar dilatation compared to primary DES and healthy controls, higher meibum reflectivity, and decreased diameters of gland orifices. Moreover, very interestingly, the authors showed increased acinar density in primary DES (Figure 4). The reduced lipid amount induces tear film hyperosmolarity, which leads to tear film instability, increased evaporation, and ocular surface inflammation [46]. A new integrated laser scanning confocal microscopy approach recently found some differences in the MGs features in patients with primary Sjogren syndrome, non-Sjogren syndrome dry eye, and MGD [32]. The pattern of inhomogeneity of the MGs walls and interstices was markedly higher in all groups of patients than in controls, with features more pronounced in primary Sjogren syndrome compared to non-Sjogren syndrome dry eye. The inhomogeneous appearance varies with the level of inflammation that is significantly higher in eyes with Sjogren syndrome, because of the autoimmune pathogenesis of this condition. The presence of confocal signs of inflammation with the absence of dilative morphologic changes supports the occurrence of an inflammatory/atrophic nonobstructive MGD in the primary Sjogren syndrome [24, 33, 48].

Ban et al. [34] studied the morphological changes of MGs in patients with dry eye due to chronic graft-versus-host disease (GVHD), a major cause of morbidity and mortality in patients undergoing allogeneic hematopoietic stem cell transplantation for hematologic malignancies. They showed that the acinar unit density and the longest and shortest diameters of MGs acini are significantly decreased. Patients with severe DES after chronic GVHD show glandular fibrosis with MGs atrophy.

LSCM was also used to evaluate MGs modification in allergic keratoconjunctival diseases, such as vernal and atopic keratoconjunctivitis (VKC, AKC), which may frequently lead to secondary dry eye [VZ, VW]. In VKC, extensive periglandular Langerhans cell infiltration, with blurred lumen contours and hyperreflective solid matter in the lumen, was described [35, 39]. Ibrahim et al. [36] reported severe MGs changes in patients with AKC, with extensive fibrotic changes much more evident than that observed in MGD. These patients present shrunken MGs with extensive periglandular fibrosis, which does not allow gland enlargement as what occurs in obstructive MGD [25].

In conclusion, in patients with dry eye, confocal microscopy revealed that the inflammation is the first and most important pathogenetic mechanism involved in the development of the MG alterations. The main findings were the increased acinar density and the reduced orifice diameter, which differentiated DES from primary or secondary MGD (Table 1). In this way, DES seems to lead to an incomplete inflammatory-induced MGD. Other particular conditions potentially leading to dry eye, such as VK, AK, and GHVD, present also various degree of periglandular fibrotic reaction, which finally causes MGs atrophy. In this wide scenario of dry eye syndromes, LSCM may help clinicians in the early diagnosis of each type of disease leading to dry eye and in monitoring the response to therapy.

### 2.4. Meibomian Glands in Ocular Allergy

AKC and VKC are two of the most common and important ocular allergies. AKC is a bilateral chronic hypersensitivity disease characterized by conjunctival papillary hypertrophy, acute and chronic conjunctivitis, keratitis and corneal ulceration, eyelid eczema, and blepharitis [49–53]. Tears proinflammatory cytokines are responsible for the conjunctival and corneal damage and also for the MG alterations. MG changes play a critical role in the development of the allergy-related dry eye [29].

**Figure 5:**
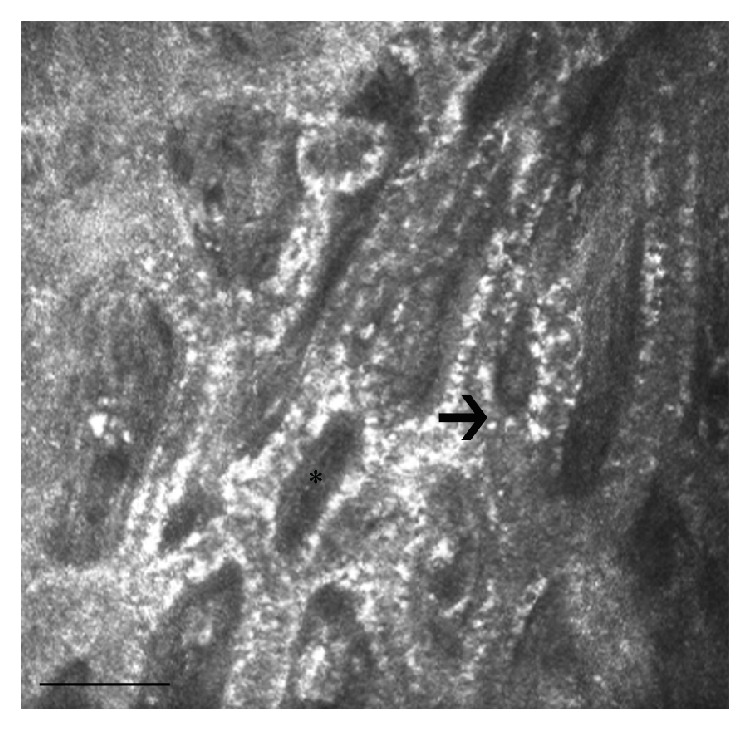
Morphological changes of MGs in a 38-year-old female patient with VKC: confocal microscopy shows extensive periglandular mononucleate cell infiltration (arrow), acinar atrophy, and ductal dilation, along with increased reflectivity of the acinar wall (asterisk). Bar represents 50 *µ*m.

VKC is a recurring seasonally inflammatory condition of the cornea and conjunctiva, characterized by giant tarsal papillae and pathological changes of the conjunctival epithelium and MGs. LSCM documented intense infiltration of eosinophils and multinucleated granulocytes in the superficial conjunctival epithelium, extensive periglandular lymphocytic cells infiltration. These inflammatory alterations lead to macroscopic modifications of MGs, represented by glandular atrophy, and ductal dilation (Figure 5). The same morphofunctional changes were described in ACK patients, where there is an important decrease in size and density of MG acinar units. MGs appear small and irregular with periglandular fibrosis and a restricted and not well-defined lumen, occluded by hyperreflective and dense meibum [16, 31, 37, 54].

In allergic patients, the fibrotic involution of MG acinar units is more extensive than that observed in obstructive MGD, where the pressure induced by the stagnating meibum induces enlargement of MG acini [29].

In summary, MG modifications in patients with ocular allergy diseases are in part different from those observed in MGD and dry eye, since the periacinar fibrosis, along with the inflammatory infiltration and the meibum stagnation, represents the main finding (Table 1). In these ocular surface diseases, probably because of the periglandular compression induced by the fibrosis and the intense inflammation, MGs seem to be unable to activate adaptive mechanisms and develop a progressive atrophy. At present, the small sample size of research studies and poor standardization of examination and interpretation are the most challenging issues.

### 2.5. Meibomian Glands in Glaucoma

In the last years, LSCM was also used to evaluate the impact of glaucoma therapy on ocular surface structures [5–7, 55], including MGs [40]. In these studies, the modifications induced by the different classes of topical medications, the impact of the preservative and active compounds, and the role of the number of daily instillations in patients in multitherapy have been investigated.

MGs presented a reduction of glandular density and area and increased reflectivity of the acinar secretion, more evident in patients treated with two or more drugs compared to patients in monotherapy. The reduced density and area were expressions of glandular loss and reduced meibum production, respectively. Higher secretion reflectivity indicated increased secretion viscosity.

**Figure 6:**
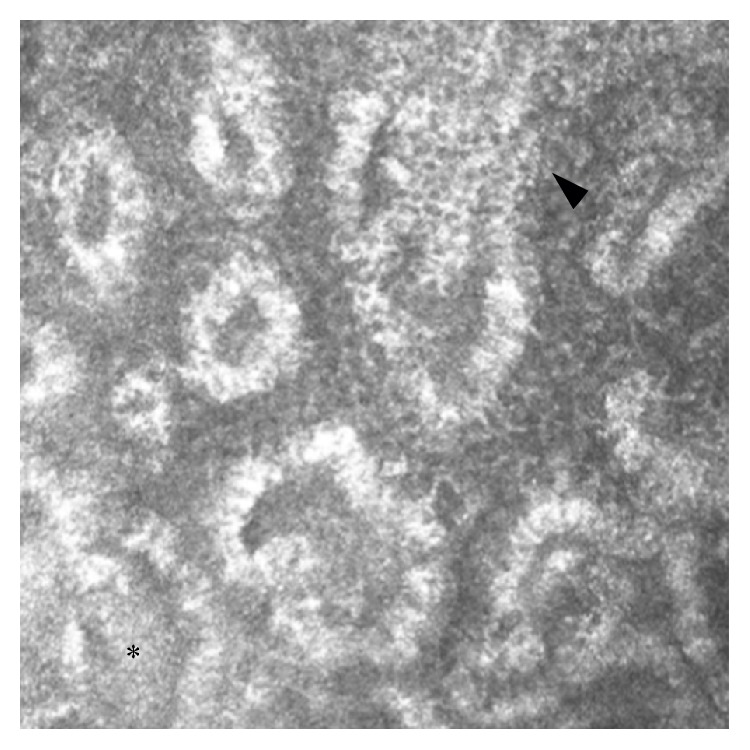
LSCM of MGs in a glaucomatous patient in multitherapy. The acinar unit density and area appeared to be reduced; MG wall and periglandular interstice presented inhomogeneous appearance, with some scattered hyperreflective punctate elements (arrowhead), and increased density of the meibum (asterisk). Bar represents 50 *µ*m.

These modifications were similar to those observed in patients with MGD, with the exception of the glandular area that appeared to be reduced in glaucoma (Figure 6). Higher secretion reflectivity could indicate increased meibum secretion viscosity.

Other aspects characterizing MGs in glaucoma are the ductal orifice dilation and the inflammation of the glandular wall and interstice, which confocal microscopy documented with the presence of punctate hyperreflective elements. The ductal orifice dilation was probably an adaptive mechanism to overcome the high secretion density and duct blockage induced by treatment.

The potential induction mechanisms are the toxicity and an inflammatory or immune-mediated response, with the inflammation probably being the first step in the cascade of glandular modifications [7, 12].

In patients in monotherapy LSCM contributed to clarifying the role of preservatives (benzalkonium chloride (BAK)) and active compounds in the acinar modifications: preserved drugs were more toxic than preservative-free (PF) formulations, with preserved prostaglandin analogues (PGA) being more toxic than preserved *β*-blockers. Therefore, BAK and PGA, with their toxic and inflammatory stimuli, play the most crucial role in the induction of glaucoma therapy-related MGD.

Very interestingly, all the microscopic modifications of MGs significantly correlated with the ocular surface disease index (OSDI) score, break-up time (BUT), and Schirmer test I (STI). These correlations indicated that MGs alterations, as assessed with LSCM, were indicators of dry eye and have a main role in development of the therapy-related ocular surface disease in patients with glaucoma. Thus, the preservation of the structural and functional integrity of MGs represents a critical challenge during medical management of glaucoma.

In conclusion, the long-term antiglaucoma therapy has a strong negative impact on MGs functionality: the iatrogenic changes of MGs, in fact, lead to meibomian gland dysfunction very similar to primary MGD, with the exception of the reduced acinar area (Table 1). It is hypothesizable that toxicity induces secondary meibomian gland dysfunction in which glands are unable to activate processes that attempt to overcome the secretion blockage. As a consequence, the acinar size decreases and MGs progressively drop out. These features are shared with CLWs, in which the mechanical trauma leads to a secondary MGD, with the exception of the reduced acinar area. One may suppose that when the inflammation plays the main role, MGs modify their features as in primary MGD, whereas when toxic or mechanic stimuli play the main role, MGs reduce also their size.

These modifications present evident clinical implications since they could strongly affect adherence and persistence of treatment.

## 3. Summary and Conclusions

Until recently, the microanatomy evaluation of the ocular surface structures was limited to the impression cytology, which is based on sampling the superficial epithelial layers. This methodology, despite being highly reproducible, does not allow exploring deep tissues and induces discomfort to patients.

The rapid diffusion of LSCM in the last decades permitted an* in vivo* biopsy of all ocular surface tissues, at different depths, and the tissue analysis at cellular and subcellular level. The meibomian gland is one of the ocular surface structures that mostly benefited from the confocal assessment, since the standard meibography can only give a macroscopic analysis of the entire gland. The possibility of imaging MGs at cellular level,* in vivo*, and in a noninvasive way permit studying these structures in the most diffuse and important ocular surface diseases such as dry eye, ocular allergy, and drug toxicity.

In this way, LSCM may allow a preclinical diagnosis of MG conditions, potentially before glands begin to malfunction. In addition, since confocal microscopy does not produce significant discomfort for patients, this technique offers the advantage to strictly monitor the disease over time, to anticipate the treatment when required, to follow the response to therapy, and to modify the therapy regimen accordingly.

Moreover, the fine microscopic assessment could also clarify the pathophysiology of these conditions. This is even more important considering that the incidence of ocular surface diseases is exponentially growing in the last years.

## Figures and Tables

**Table 1:** Summary of the most important confocal signs for each disease affecting MGs.

Conditions	LSCM characteristics	References
Aging	MG acinar unit density and diameter reduction; increase of the acinar wall inhomogeneity	[13, 15, 16, 24, 25]

MGD	MG acinar unit density reduction; larger acinar unit diameter; duct dilation	[26–31]

Dry eye	MG acinar unit density and diameter reduction; inhomogeneous appearance of the MG walls and interstices; extensive periglandular Langerhans cell infiltration	[13, 16, 24, 25, 32–35]

Ocular allergy	Eosinophils and multinucleated granulocytes in the superficial conjunctival epithelium; periglandular lymphocytic cell infiltration; glandular atrophy and acinar and ductal dilatation	[13, 16, 24, 25, 36–39]

Glaucoma	MG acinar unit density and area reduction; increased reflectivity of the acinar secretion; ductal orifice dilation; inhomogeneity of MG interstice and wall	[9, 40]

## References

[1] Knop E., Knop N., Millar T., Obata H., Sullivan D. A. (2011). The international workshop on meibomian gland dysfunction: report of the subcommittee on anatomy, physiology, and pathophysiology of the meibomian gland. *Investigative Ophthalmology and Visual Science*.

[2] Mishima S., Maurice D. M. (1961). The oily layer of the tear film and evaporation from the corneal surface. *Experimental Eye Research*.

[3] Tiffany J. M. (1987). The lipid secretion of the meibomian glands. *Advances in Lipid Research*.

[4] Jester J. V., Rife L., Nii D., Luttrull J. K., Wilson L., Smith R. E. (1982). In vivo biomicroscopy and photography of meibomian glands in a rabbit model of meibomian gland dysfunction. *Investigative Ophthalmology and Visual Science*.

[5] Mastropasqua R., Fasanella V., Pedrotti E. (2014). Trans-conjunctival aqueous humor outflow in glaucomatous patients treated with prostaglandin analogues: an in vivo confocal microscopy study. *Graefe's Archive for Clinical and Experimental Ophthalmology*.

[6] Agnifili L., Carpineto P., Fasanella V. (2012). Conjunctival findings in hyperbaric and low-tension glaucoma: an in vivo confocal microscopy study. *Acta Ophthalmologica*.

[7] Mastropasqua L., Agnifili L., Fasanella V. (2013). Conjunctival goblet cells density and preservative-free tafluprost therapy for glaucoma: an in vivo confocal microscopy and impression cytology study. *Acta Ophthalmologica*.

[8] Mastropasqua L., Nubile M., Lanzini M. (2013). Morphological modification of the cornea after standard and transepithelial corneal cross-linking as imaged by anterior segment optical coherence tomography and laser scanning in vivo confocal microscopy. *Cornea*.

[9] Agnifili L., Fasanella V., D'Aguanno S. (2013). Shotgun proteomics reveals specific modulated protein patterns in tears of patients with primary open angle glaucoma naïve to therapy. *Molecular BioSystems*.

[10] Mastropasqua R., Agnifili L., Fasanella V. (2015). Corneoscleral limbus in glaucoma patients: in vivo confocal microscopy and immunocytological study. *Investigative Opthalmology & Visual Science*.

[11] Carpineto P., Agnifili L., Nubile M. (2011). Conjunctival and corneal findings in bleb-associated endophthalmitis: an in vivo confocal microscopy study. *Acta Ophthalmologica*.

[12] Mastropasqua L., Agnifili L., Mastropasqua R. (2014). In vivo laser scanning confocal microscopy of the ocular surface in glaucoma. *Microscopy and Microanalysis*.

[13] Villani E., Baudouin C., Efron N. (2014). In vivo confocal microscopy of the ocular surface: from bench to bedside. *Current Eye Research*.

[14] Efron N., Al-Dossari M., Pritchard N. (2009). In vivo confocal microscopy of the palpebral conjunctiva and tarsal plate. *Optometry and Vision Science*.

[15] Wei A., Hong J., Sun X., Xu J. (2011). Evaluation of age-related changes in human palpebral conjunctiva and meibomian glands by in vivo confocal microscopy. *Cornea*.

[16] Villani E., Canton V., Magnani F., Viola F., Nucci P., Ratiglia R. (2013). The aging meibomian gland: an in vivo confocal study. *Investigative Ophthalmology and Visual Science*.

[17] Sullivan D. A., Sullivan B. D., Ullman M. D. (2000). Androgen influence on the meibomian gland. *Investigative Ophthalmology and Visual Science*.

[18] Sullivan B. D., Evans J. E., Cermak J. M., Krenzer K. L., Dana M. R., Sullivan D. A. (2002). Complete androgen insensitivity syndrome: effect on human meibomian gland secretions. *Archives of Ophthalmology*.

[19] Auw-Haedrich C., Feltgen N. (2003). Estrogen receptor expression in meibomian glands and its correlation with age and dry-eye parameters. *Graefe's Archive for Clinical and Experimental Ophthalmology*.

[20] Hom M. M., Martinson J. R., Knapp L. L., Paugh J. R. (1990). Prevalence of meibomian gland dysfunction. *Optometry and Vision Science*.

[21] Bodineau A., Folliguet M., Séguier S. (2009). Tissular senescence and modifications of oral ecosystem in the elderly: risk factors for mucosal pathologies. *Current Aging Science*.

[22] Rocha E. M., Alves M., Rios J. D., Dartt D. A. (2008). The aging lacrimal gland: changes in structure and function. *Ocular Surface*.

[23] Agnifili L., Mastropasqua R., Fasanella V. (2014). In vivo confocal microscopy of conjunctiva-associated lymphoid tissue in healthy humans. *Investigative Ophthalmology and Visual Science*.

[24] Villani E., Magnani F., Viola F. (2013). In vivo confocal evaluation of the ocular surface morpho-functional unit in dry eye. *Optometry and Vision Science*.

[25] Villani E., Mantelli F., Nucci P. (2013). In-vivo confocal microscopy of the ocular surface: ocular allergy and dry eye. *Current Opinion in Allergy and Clinical Immunology*.

[26] Kobayashi A., Yoshita T., Sugiyama K. (2005). In vivo findings of the bulbar/palpebral conjunctiva and presumed meibomian glands by laser scanning confocal microscopy. *Cornea*.

[27] Messmer E. M., Mackert M. J., Zapp D. M., Kampik A. (2006). In vivo confocal microscopy of normal conjunctiva and conjunctivitis. *Cornea*.

[28] Messmer E. M., Torres Suárez E., Mackert M. I., Zapp D. M., Kampik A. (2005). In vivo confocal microscopy in blepharitis. *Klinische Monatsblatter fur Augenheilkunde*.

[29] Matsumoto Y., Sato E. A., Ibrahim O. M. A., Dogru M., Tsubota K. (2008). The application of in vivo laser confocal microscopy to the diagnosis and evaluation of meibomian gland dysfunction. *Molecular Vision*.

[30] Ibrahim O. M. A., Matsumoto Y., Dogru M. (2010). The efficacy, sensitivity, and specificity of in vivo laser confocal microscopy in the diagnosis of meibomian gland dysfunction. *Ophthalmology*.

[31] Ibrahim O. M. A., Matsumoto Y., Dogru M. In vivo confocal microscopy evaluation of meibomian gland dysfunction in atopic-keratoconjunctivitis patients.

[32] Villani E., Ceresara G., Beretta S., Magnani F., Viola F., Ratiglia R. (2011). In vivo confocal microscopy of meibomian glands in contact lens wearers. *Investigative Ophthalmology and Visual Science*.

[33] Villani E., Beretta S., De Capitani M., Galimberti D., Viola F., Ratiglia R. (2011). In vivo confocal microscopy of meibomian glands in Sjögren's syndrome. *Investigative Ophthalmology and Visual Science*.

[34] Ban Y., Ogawa Y., Ibrahim O. M. A. (2011). Morphologic evaluation of Meibomian glands in chronic graft-versus-host disease using in vivo laser confocal microscopy. *Molecular Vision*.

[35] Le Q., Hong J., Zhu W., Sun X., Xu J. (2011). In vivo laser scanning confocal microscopy of vernal keratoconjunctivitis. *Clinical and Experimental Ophthalmology*.

[36] Ibrahim O. M. A., Matsumoto Y., Dogru M. (2012). In vivo confocal microscopy evaluation of meibomian gland dysfunction in atopic-keratoconjunctivitis patients. *Ophthalmology*.

[37] Villani E., Strologo M. D., Pichi F. (2015). Dry eye in vernal keratoconjunctivitis: a cross-sectional comparative study. *Medicine (Baltimore)*.

[38] Villani E., Garoli E., Canton V., Pichi F., Nucci P., Ratiglia R. (2014). Evaluation of a novel eyelid-warming device in meibomian gland dysfunction unresponsive to traditional warm compress treatment: an in vivo confocal study. *International Ophthalmology*.

[39] Wei Q., Le Q., Hong J., Xiang J., Wei A., Xu J. (2015). In vivo confocal microscopy of meibomian glands and palpebral conjunctiva in vernal keratoconjunctivitis. *Indian Journal of Ophthalmology*.

[40] Agnifili L., Fasanella V., Costagliola C. (2013). In vivo confocal microscopy of meibomian glands in glaucoma. *British Journal of Ophthalmology*.

[41] Stanek S. (2000). Meibomian gland status comparison between active duty personnel and U.S. veterans. *Military Medicine*.

[42] Matsumoto Y., Shigeno Y., Sato E. A. (2009). The evaluation of the treatment response in obstructive meibomian gland disease by in vivo laser confocal microscopy. *Graefe's Archive for Clinical and Experimental Ophthalmology*.

[43] Qazi Y., Kheirkhah A., Blackie C. (2015). In vivo detection of clinically non-apparent ocular surface inflammation in patients with meibomian gland dysfunction-associated refractory dry eye symptoms: a pilot study. *Eye*.

[44] Cunniffe M. G., Medel-Jiménez R., González-Candial M. (2011). Topical antiglaucoma treatment with prostaglandin analogues may precipitate meibomian gland disease. *Ophthalmic Plastic and Reconstructive Surgery*.

[45] Arita R., Itoh K., Maeda S. (2012). Comparison of the long-term effects of various topical antiglaucoma medications on meibomian glands. *Cornea*.

[46] Arita R., Itoh K., Maeda S. (2012). Effects of long-term topical anti-glaucoma medications on meibomian glands. *Graefe's Archive for Clinical and Experimental Ophthalmology*.

[47] International Dry Eye WorkShop (2007). The definition and classification of dry eye disease: report of the definition and classification subcommittee of the international dry eye workshop. *The Ocular Surface*.

[48] Alhatem A., Cavalcanti B., Hamrah P. (2012). In vivo confocal microscopy in dry eye disease and related conditions. *Seminars in Ophthalmology*.

[49] Bacon A. S., Tuft S. J., Metz D. M. (1993). The origin of keratopathy in chronic allergic eye disease: a histopathological study. *Eye*.

[50] Dogru M., Katakami C., Nakagawa N., Tetsumoto K., Yamamoto M. (1998). Impression cytology in atopic dermatitis. *Ophthalmology*.

[51] Tuft S. J., Kemeny D. M., Dart J. K. G., Buckley R. J. (1991). Clinical features of atopic keratoconjunctivitis. *Ophthalmology*.

[52] Forster C. S., Calonge M. (1990). Atopic keratoconjunctivitis. *Ophthalmology*.

[53] Hogan M. J. (1952). Atopic keratoconjunctivitis. *Transactions of the American Ophthalmological Society*.

[54] Dogru M., Okada N., Asano-Kato N. (2005). Atopic ocular surface disease: implications on tear function and ocular surface mucins. *Cornea*.

[55] Mastropasqua L., Agnifili L., Mastropasqua R., Fasanella V. (2013). Conjunctival modifications induced by medical and surgical therapies in patients with glaucoma. *Current Opinion in Pharmacology*.

